# Second hand smoke and COPD: lessons from animal studies

**DOI:** 10.3389/fphys.2013.00030

**Published:** 2013-02-27

**Authors:** Monica P. Goldklang, Sarah M. Marks, Jeanine M. D'Armiento

**Affiliations:** Department of Medicine, Columbia UniversityNew York, NY, USA

**Keywords:** emphysema, COPD, animal models, cigarette smoke, inflammation, apoptosis, proteases

## Abstract

Exposure to second hand smoke is a major cause of chronic obstructive pulmonary disease (COPD) in the non-smoker. In this review we explore the use of animal smoke exposure models and their insight into disease pathogenesis. The methods of smoke exposure, including exposure delivery systems, are described. Key findings from the acute and chronic smoke exposure models are outlined, including descriptions of the inflammation processes, proteases involved, oxidative stress, and apoptosis. Finally, alternatives to rodent models of lung disease are presented.

## Introduction

Chronic obstructive pulmonary disease (COPD) is a major cause of disability and death worldwide, with a prevalence estimated at 10% of the general population and up to 50% in heavy smokers (Rennard and Vestbo, 2006). As the incidence of COPD rises, associated health care costs will increase as well. Exposure to tobacco smoke is a key etiologic agent of this disease, which is characterized by progressive airflow limitation with an abnormal inflammatory response in the small airways and alveoli. The inflammation induced by cigarette smoke leads to an increase in protease production, a major contributor to the lung destruction seen in emphysema (Churg et al., 2008).

For the past 50 years emphysematous lung destruction was believed to arise from an imbalance between proteases and anti-proteases. This theory was originally formulated after it was noted that smokers with a congenital deficiency of alpha-1-antitrypsin (α_1_-AT), leading to unopposed neutrophil elastase activity, had an increased incidence of emphysema (Laurell and Erickson, 1963). Imbalances between matrix metalloproteinases (MMPs) and their endogenous inhibitors, tissue inhibitors of metalloproteinases (TIMPs), have been identified in emphysema tissues (Finlay et al., 1997; Hautamaki et al., 1997; Ohnishi et al., 1998; Imai et al., 2001; Houghton et al., 2006; Lee et al., 2009; Silverman and Sandhaus, 2009). MMPs are a family of structurally related zinc-dependent endopeptidases involved in the degradation and remodeling of the extracellular matrix during a variety of important physiological and pathological processes (Visse and Nagase, 2003) including embryological development, angiogenesis, and wound healing (Chang and Werb, 2001). Studies in our laboratory, and others, have shown that cigarette smoke directly induces expression of MMPs in resident lung cells (Mercer et al., 2004; Seagrave et al., 2004; Valenca et al., 2004).

Exposure to second hand tobacco smoke is a major risk factor for the development of COPD in non-smokers. Based upon plasma cotinine levels, a non-smoker living with a smoker has a smoke exposure of approximately 1% of that from an individual actively smoking 20 cigarettes a day (Thompson et al., 1990; Law and Hackshaw, 1996). Despite the low level of exposure, those exposed to second hand smoke exhibit increased levels of elastin degradation products in the serum (Slowik et al., 2011); these degradation products are chemotactic for macrophages in the lung, promoting ongoing inflammation, and lung degradation (Houghton et al., 2006). Genetic factors influence the development of COPD in smokers (Silverman, 2006) and likely impact the inflammatory response to second hand smoke. It should be noted that in the developing world, the burning of biomass fuels for cooking and heating has been implicated in the development of COPD in non-smoking adults (Da Silva et al., 2012).

COPD is manifested by several different pathologies: emphysema, small airway remodeling, chronic bronchitis, and pulmonary hypertension (Wright et al., 2008). Frequent COPD exacerbations lead to further decline in lung function in patients with moderate to severe disease (Donaldson et al., 2002). With variation in pathologies and clinical manifestations, no single animal model can serve as a surrogate to the human disease. The structural and mechanical changes in animal models of COPD can be quite mild, but information regarding second hand smoke exposure can be gained through examination of the appropriate susceptible species and mouse strains. It is essential that researchers understand the differences in smoke-exposure animal models in order to better understand the effects of second hand smoke in humans.

## Methods of smoke exposure in animal models of emphysema

There are two main systems used for smoke exposure of rodents, nose-only smoke exposure machines and whole body exposure systems (Coggins, 1998). Nose-only smoke systems offer the advantages of more controlled dosage and exposure as well as smoke delivery only to the respiratory system. However, this method of nasal smoke exposure also involves prolonged whole-body restraint, a known stressor for mice (van Eijl et al., 2006). The metal nosepieces utilized in the restraining system have also been associated with severe hypothermia (van Eijl et al., 2006).

Whole body smoke exposure systems offer several advantages. Rodents do not require restraint for smoking, but rather are housed in their usual cages and bedding. They have free access to food and water for the duration of smoke exposure. In addition, the setup of most whole body smoke exposure systems allows for a large number of rodents and small animals to undergo simultaneous smoke exposure and therefore it is less labor intensive than nose-only systems. On the other hand, the whole body smoke exposure system is less controlled than the nose-only system; though, measurement of total particulate matter can aid in ensuring steady smoke exposure during animal experiments. In addition, in whole body smoke exposure systems animals develop deposits of nicotine and tar on the fur, which can be ingested through usual grooming behaviors (Mauderly et al., 1989; Wright et al., 2008). Regardless of the chosen method of smoke exposure, the quality of smoke exposure can be measured through carboxyhemoglobin and serum cotinine levels (Wright et al., 2008).

Cigarette smoke, delivered either by nose-only or whole body exposure systems, is composed of both mainstream and side stream smoke. Mainstream smoke is derived directly from the cigarettes during the inhalation phase or puff of the smoke machine. Side stream smoke refers to the smoke generated from the lit end of a cigarette in between puffs, and is the main component of second hand smoke. In an evaluation of unpublished work from Phillip Morris in the 1980's, fresh side stream smoke is approximately four times more toxic per gram total particulate matter than mainstream smoke (Schick and Glantz, 2005). Aged side stream smoke (over 10 s old) increases the toxicity four-fold as compared to fresh side stream smoke (Schick and Glantz, 2006). All of these findings are particularly relevant to animal smoke exposure systems, which utilize both aged side stream and mainstream smoke.

## Lessons from chronic and acute smoke exposure studies

In order to develop structural and physiological changes in the lung consistent with emphysema, investigators undertake long-term smoke exposure studies. Variation between investigators exists, but it is generally accepted that in the mouse model, at least 6 months of smoke exposure is required to produce structural and physiological changes consistent with emphysema (Churg et al., 2008). The exception to this is the highly susceptible A/J strain, which can develop emphysematous changes, as measured by mean linear intercept, after only 4 months of smoke exposure (Braber et al., 2011). The literature describing studies on acute smoke exposure are much more variable with regards to the exact duration of exposure, ranging from hours to weeks. Common to all smoke exposure studies in animals is the development of pulmonary inflammation. However, the inflammatory cell characteristics vary by the chosen method of exposure, species, and within mice based on strains (van der Vaart et al., 2004).

In both the chronic and acute smoke exposure models, an increase in the number of alveolar macrophages is induced by smoke in the bronchoalveolar lavage (BAL) fluid and lung tissue (van der Vaart et al., 2004); increases in neutrophil counts are more variable. Increased alveolar macrophages in BAL fluid can be identified immediately following smoke exposure (Ortega et al., 1992) and persist throughout the duration of the exposure. Despite the increase in alveolar macrophage numbers, alveolar macrophages from 8-week smoke-exposed mice demonstrate an attenuation of the inflammatory signaling pathways associated with bacterial and viral infections, including TNF-α and IL-6 (Gaschler et al., 2008) and exhibit an impaired phagocytic activity immediately following smoke exposure (Ortega et al., 1992).

Early chronic smoke exposure studies in mice focused mainly on the carcinogenic properties of tobacco smoke. Initial studies of non-malignant pulmonary abnormalities caused by chronic smoke exposure focused on alterations in the inflammatory response. Findings included peribronchiolar and perivascular accumulations of lymphocytes and macrophages (Matulionis, 1984), with increased macrophage size due to lysosomal abnormalities (Matulionis and Simmerman, 1985). Chronic smoke exposure was also found to reduce antigen-specific T-cell proliferative response in pulmonary associated lymph nodes (Chang et al., 1990) as well as the phagocytic index of macrophages (Ortega et al., 1992). Although structural changes in the mouse lung occur after long-term smoke exposure, it is important to note that the changes in mice following chronic smoke exposure are still only mild as measured by mean linear intercept or lung compliance (Foronjy et al., 2005, 2006; Churg et al., 2008).

With the development of genetically modified animals, researchers have been able to better define the contribution of isolated pathways in emphysema pathogenesis, including the interstitial collagenase MMP-1 (D'Armiento et al., 1992). The strength of these animal studies was extended when follow-up studies demonstrated that indeed MMP-1 expression was increased in the lung parenchyma of patients with emphysema (Imai et al., 2001) and that cigarette smoke directly induced expression of MMP-1 in pulmonary small airway epithelial cells. Building upon the long known association between emphysema development in smokers and congenital deficiency of α_1_-AT (Laurell and Erickson, 1963), Shapiro and colleagues established the critical role of MMP-12 (macrophage elastase) in emphysema development (Hautamaki et al., 1997). Following the development of the MMP-12 knockout mouse (Shipley et al., 1996), the group demonstrated that smoke-exposed mice deficient in MMP-12 do not develop parenchymal changes consistent with emphysema (Hautamaki et al., 1997). In addition to the role of MMP-12 in chronic smoke exposure, MMP-12 is also involved in the acute inflammatory response by augmenting TNF-alpha release from macrophages, causing neutrophil influx and endothelial activation (Churg et al., 2003). Elastin degradation products, by-products of MMP-12 activity, promote inflammation and ongoing lung degradation (Houghton et al., 2006).

Other laboratories have built upon the MMP-12 work, documenting the importance of additional MMPs in emphysema pathogenesis (Shapiro et al., 2003; Seagrave et al., 2004; March et al., 2006; Valenca et al., 2006; Foronjy et al., 2008; Vecchio et al., 2010). Our laboratory has demonstrated that the transgenic overexpression of MMP-9 in macrophages causes spontaneous adult-onset emphysema due to elastin degradation (Foronjy et al., 2008). In contrast, Atkinson and colleagues have shown that the MMP-9 deficient mouse still develops smoke-induced emphysema (Atkinson et al., 2011). It is likely that in MMP-9 deficient mice, the presence of other proteases, including MMP-12, are sufficient to destroy the lung; yet, MMP-9 potentially plays a role in the destructive process in combination with other proteases.

Beyond the protease/antiprotease hypothesis, transgenic mice have shed light into additional pathways critical in emphysema pathogenesis. A substantial quantity of work has been performed examining the effect of cigarette smoke on inflammatory pathways. TNF-alpha is a key pro-inflammatory factor implicated in emphysema pathogenesis. TNF-alpha receptor 2 plays a critical role in this proinflammatory pathway, as mice deficient in the receptor have both attenuated pulmonary inflammation and emphysema development in the setting of long-term smoke exposure (D'Hulst et al., 2006). The pro-inflammatory cytokine IFN-gamma is a potent stimulator of MMP-9 and CCR5 ligands, the expression of which ultimately results in DNA damage, apoptosis, and emphysema (Ma et al., 2005). The inflammatory cell surface receptor CX3CR1 is upregulated by cigarette smoke exposure; expression of CX3CR1 results in the recruitment of other CX3CR1 positive cells, mainly macrophages and T lymphocytes, amplifying the inflammatory response to cigarette smoke exposure (McComb et al., 2008). Other pathways implicated in modulation of the inflammatory response in smoke exposure include protein phosphatase 2A (PP2A) (Wallace et al., 2012), forkhead box class O 3a (FOXO3) (Hwang et al., 2011), CCR6 (Bracke et al., 2006), and endothelial monocyte-activating protein 2 (EMAPII) (Clauss et al., 2011).

Oxidative stress is another key mediator in emphysema pathogenesis and the pathways of lung injury (Yao and Rahman, 2011; Rahman, 2012). The acute smoke exposure model is well-suited to examine cellular oxidative stress and mechanisms leading to increased inflammation (Valenca et al., 2008; Richens et al., 2009; Gould et al., 2010; Geraghty et al., 2011b; Lu et al., 2011). Within 6 h of smoke exposure, evidence of oxidative stress can be detected within the pulmonary vascular endothelium (Lu et al., 2011). Many different pathways alter the levels of oxidative stress within the lung following smoke exposure. Superoxide dismutase (SOD) is an enzyme that catalyzes the breakdown of superoxide radicals into oxygen and hydrogen peroxide. Our laboratory first demonstrated a protective role of antioxidants when transgenic mice expressing elevated levels of SOD in the lung were shown to be protected from the development of emphysema through attenuation of the inflammatory response, particularly neutrophil inflammation after smoke exposure and intratracheal elastase administration (Foronjy et al., 2006). The loss of antioxidants were shown by Rahman and colleagues to lead to impaired lung function and exercise capacity following chronic cigarette smoke exposure in mice lacking SOD3 (Yao et al., 2010). Moreover, overexpression of SOD3 as well as exogenous administration of SOD analogues attenuated emphysema development (Yao et al., 2010) in smoke exposure models. SOD appears to provide protective benefits against oxidative stress mediated fragmentation of the extracellular matrix (Yao et al., 2010). Another interesting area of research in emphysema relates to endoplasmic reticulum (ER) stress. In the setting of acute smoke exposure, inflammatory cells, predominately macrophages, develop ER stress through CHOP induction by cigarette smoke, but during *in vivo* long-term smoke exposure, the ER stress response is reduced (Geraghty et al., 2011b). The finding of decreased ER stress in long-term smoke exposed mice is analogous to the human disease, where in human emphysematous lung tissue, there is a decrease in markers of ER stress (Korfei et al., 2008).

Other critical pathways modulating oxidative stress that were shown to be important in the smoke exposure model include the mTOR pathway, where smoke exposure upregulates the mTOR inhibitor and stress related protein Rtp801, amplifying oxidative stress, NF-kappa-B activation and subsequent alveolar inflammation and alveolar septal apoptosis (Yoshida et al., 2010). Nrf2, a regulator of the antioxidant pathway, also plays a major role in determining susceptibility to smoke-induced emphysema by upregulating antioxidants and thereby decreasing inflammation (Rangasamy et al., 2004, 2009; Iizuka et al., 2005; Gebel et al., 2010). Additionally, the TGF-β signaling pathway influences oxidative stress in the setting of smoke exposure; antagonism of this pathway with the angiotensin receptor type 1 blocker losartan decreased oxidative stress, inflammation and extracellular matrix remodeling (Podowski et al., 2012). Furthermore, the class III histone/protein deacetylase SIRT1 regulates cigarette smoke-exposure mediated proinflammatory signaling through NF-kappa-B and levels are decreased in rats following smoke exposure (Yang et al., 2007). SIRT1 levels are also decreased in both macrophages and the lung tissue of smokers and patients with COPD (Rajendrasozhan et al., 2008). Activation of the SIRT1 pathway *in vitro* with resveratrol decreases proinflammatory cytokine release (Yang et al., 2007; Arunachalam et al., 2010), and both genetic overexpression and pharmacologic activation of SIRT1 protects against smoke-induced emphysema via a FOXO3-mediated reduction of cellular senescence independent of the inflammatory effects (Yao et al., 2012).

The pattern recognition receptor toll-like receptor-4 (TLR4), mediated through the NADPH oxidase (NOX) pathway, is another important component in the oxidative stress pathway critical in emphysema development. Lee and colleagues have shown that deficiency of TLR4 leads to upregulation of NOX2 in lungs and endothelial cells, causing increased oxidant generation and elastolytic activity within the parenchyma (Zhang et al., 2006). TLR4 deficiency also promotes apoptosis and autophagy in the setting of smoke exposure, with increased airspace enlargement as compared to smoked wild-type mice (An et al., 2012), suggesting a protective benefit to TLR4 expression in the lung. However, TLR4 activation by cigarette smoke also has negative effects within the lung. Lipopolysaccharide (LPS) is a component of cigarette smoke as well as a known TLR4 ligand; activation of TLR4 leads to IL-1 production, IL-1 receptor 1 signaling, and downstream inflammation (Doz et al., 2008). Our group has demonstrated that TLR4 is upregulated within the lungs of both smoke-exposed mice and rabbits, and have documented increased signaling through TLR4 in human emphysematous lung tissue (Geraghty et al., 2011a). When MMP-1 is present, as is seen in both human emphysema as well as in the smoke-exposed rabbit, TLR4 activation by cigarette smoke ultimately leads to expression of the collagenase MMP-1 (Geraghty et al., 2011a). In addition to the smoking model of emphysema, hyperlipidemia alone, as is seen in the ApoE knockout mouse fed a high fat diet, is sufficient to cause peribronchial and perivascular inflammation through TLR4 activation leading to emphysema development (Goldklang et al., 2012), suggesting a new mechanism linking emphysema and cardiovascular disease. The role that this specific pathway plays in smoke exposure induced emphysema remains to be determined in future studies.

Finally, apoptosis is an additional critical process in emphysema pathogenesis that has been investigated in the smoke exposure model. Apoptosis is a mechanism of programmed cell death that occurs during normal lung morphogenesis, but can also be triggered by multiple stimuli that are pertinent in emphysema development, including loss of contact with the extracellular matrix, reduction in VEGF signaling, induction by immune cells, and various stresses including oxidative stress (Morissette et al., 2009). As mentioned above, the TLR4 pathway modulates both cellular apoptosis and autophagy in response to chronic cigarette smoke exposure (An et al., 2012). The ceramide pathway, modulated by neutral sphingomyelinase 2 (nSMase2) also modulates cigarette smoke related epithelial cell apoptosis. nSMase is upregulated in the lung following smoke exposure, while knockdown of nSMase with a heterozygous mouse decreases smoke related ceramide production and apoptosis (Filosto et al., 2011). Other implicated pathways in smoke related emphysema include the TGF-β signaling pathway via SMAD-3 (Farkas et al., 2011), cytokine IL-6 (Ruwanpura et al., 2011), VEGF (Kasahara et al., 2000; Tuder et al., 2003b), and autophagy via early growth response-1 (Egr-1) and the protein microtubule-associated protein 1 light chain-3B (LC3B) (Chen et al., 2008, 2010). In summary, there are numerous pathways involved in emphysema pathogenesis. Utilizing transgenically modified mouse models and pharmacological interventions, there have been significant advances in the understanding of human disease pathogenesis and treatment.

## The development of emphysema is mouse strain dependent

One of the most important considerations in smoke exposure studies is understanding that the development of emphysema in a chronic smoke exposure model, as measured by morphometry and lung compliance, is mouse strain dependent (Guerassimov et al., 2004; Foronjy et al., 2005). In a comprehensive study performed by Guerassimov and colleagues, NZWLac/J, C57BL6/J, A/J, SJ/L, and AKR/J strains underwent 6 months of cigarette smoke exposure. In morphometric analysis of emphysema, the most impressive increase in mean linear intercept was observed in the AKR/J strain (38% increase as compared to control mice), with more modest increases seen in the A/J, SJ/L and C57BL6/J mice (Guerassimov et al., 2004). The NZWLac/J mice did not develop any increase in mean linear intercept (11% decrease as compared to control mice) (Guerassimov et al., 2004). Furthermore, despite detectible, albeit modest, alterations in lung morphometry, there is not necessarily a correlation with alterations in lung mechanics (Guerassimov et al., 2004; Foronjy et al., 2005).

A difference in the inflammatory response explains at least some of the variations between mouse strains in response to chronic smoke exposure. Again, the AKR/J mice demonstrate smoke-induced increases in macrophages, PMNs, and T cells with a Th1 cytokine predominance; increased alveolar macrophages were also noted in the C57BL6/J and NZWLac/J mice (Foronjy et al., 2005). *In vitro* analysis of alveolar macrophages from susceptible (C57BL6/J) and resistant (ICR) strains demonstrates that alveolar macrophages from susceptible mice release increased proinflammatory cytokines and MMPs when exposed to cigarette smoke, and have a higher baseline production of reactive oxygen species as compared to those obtained from resistant mice (Vecchio et al., 2010). The pro-inflammatory early growth response gene-1 (Egr-1) may provide some insight into strain variation. Egr-1 induction by cigarette smoke varies by strain, with marked increase in the emphysema-susceptible AKR/J mice as compared to the relatively resistant NZWLac/J strain (Reynolds et al., 2006).

It is important to understand that dissimilar to what is seen in the human disease, in the mouse model of smoke-induced emphysema, smoking cessation is accompanied by a reparative response (Braber et al., 2011). Within 1 week of smoking cessation, there is a partial normalization of MMP-2 and MMP-9 levels in BAL fluid (Seagrave et al., 2004), though lymphocyte abnormalities including CD8 T cell oligoclonal expansions persist (Seagrave et al., 2004; Motz et al., 2008). It is likely that the combination of altered inflammatory response and cytokine profiles are responsible for the variations between strains in structural and functional measures of emphysema as well as for the reparative response seen upon smoking cessation. However, understanding the limitations of the mouse models has led our group and others to investigate alternative animal models of emphysema.

## Smoke exposure using alternative animal species

While mice are the most widely utilized animal species for smoke exposure studies, the mouse model of emphysema has several limitations. Mice do not express the same proteolytic repertoire upon smoke exposure as humans. Mice do not even have the gene for MMP-1, a key protease implicated in human emphysema pathogenesis (D'Armiento et al., 1992). As stated above, mice develop only mild structural and mechanical changes to the lung following smoke exposure (Foronjy et al., 2005). In addition, both mice and rats undergo alveolar development entirely post-natally (Massaro and Massaro, 2007). For these reasons and others, investigators have long been interested in identifying alternative animal models of emphysema that more closely mimic the human disease process.

As opposed to rodents, the guinea pig model offers several advantages that aid in the study of the effects of smoke exposure. Guinea pigs begin lung alveolarization at birth, and exhibit a progressive increase in air space size until 18 months of age (Wright and Churg, 1990). The emphysema that guinea pigs develop is easily recognizable (Golovatch et al., 2009), with noted small airway remodeling and goblet cell metaplasia that is absent in mouse exposure models (Wright and Sun, 1994; Wright et al., 2007). Guinea pigs develop airway inflammation, with increased alveolar macrophages as well as activation of the MAP kinases ERK and JNK, as is seen in the human disease state (Golovatch et al., 2009). In addition, as opposed to mice, guinea pigs can develop significant pulmonary arterial hypertension after only 1 month of smoke exposure (Wright et al., 2006). Most interestingly, in guinea pigs the development of emphysema is cathepsin K dependent, as opposed to a metalloproteinase or elastase driven lung destructive process, and while extracellular matrix changes similar to those in the human disease were observed in the guinea pig smoke exposure model, there were no noted alterations in collagenolytic MMPs (Golovatch et al., 2009).

Furthermore, guinea pigs differ from mice in their reparative responses following smoke exposure. While all-trans retinoic acid treatment has proven to be useful in reversal of emphysematous changes of alveolar structures in mice and rats (Massaro and Massaro, 1997; Belloni et al., 2000), it does not produce the same protective effects in smoke-exposed guinea pigs (Meshi et al., 2002). Another major reparative response following smoke exposure is the increase in matrix and structural cells in the small airways and intrapulmonary arteries, but not in the alveolar wall. In both mice and guinea pigs, increased deposition of collagen in the airway has been noted after chronic smoke exposure (Churg et al., 2006, 2007). It is theorized that an increase in apoptosis in emphysematous lungs could explain this failure to repair in certain strains of mice, rats (Kasahara et al., 2000; Tuder et al., 2003a), and humans (Kasahara et al., 2001). However, our group has shown that certain strains of mice and guinea pigs do not develop increased apoptosis with smoke exposure and emphysema (Foronjy et al., 2005; Golovatch et al., 2009).

The rabbit smoke exposure model is another alternative to the rodent models. Rabbits express MMP-1 and therefore the macrophages express a protease repertoire similar to humans (Fukuda et al., 2000). Studies have not only demonstrated the presence of MMP-1 in the rabbit, but specifically MMP-1 expression during lung development is similar to human lung development (Fukuda et al., 2000). Our group has recently demonstrated that the smoke-exposed rabbit model exhibits the activation of similar mechanistic pathways following smoke exposure as is seen in the human disease (Geraghty et al., 2011a). Since it is well-documented that MMP-1 is fundamental to emphysema pathogenesis, the rabbit is the ideal species to examine when exploring the MMP-1 regulated lung destruction and determining if pharmacological inhibition of MMP-1 is a feasible approach to the treatment of emphysema.

Rabbits, similar to guinea pigs, differ from mice in their reparative response to smoke exposure. In a study of rabbits exposed to pine wood smoke, Thorning and colleagues document reparative responses that include intact epithelial basal lamina acting as a substratum for proliferating reparative epithelial cells (Thorning et al., 1982). However, more recent studies have shown that as opposed to mice the smoke-exposed rabbit does not exhibit reversal of emphysematous changes after all-trans-retinoic acid treatment (Nishi et al., 2003).

Both the rabbit and guinea pig models offer the specific advantages over the rodent model as outlined above, but the disadvantages cannot go without mention. The large size and lack of available reagents in rabbits and guinea pigs limit the ease of experimentation. The high costs associated with purchase, housing, and smoke exposure in rabbits are practical deterrents to their use. Furthermore, the lung destruction seen in the smoke-exposed guinea pig model is cathepsin dependent, but cathepsins do not play as significant a role in emphysema pathogenesis in humans as MMPs (Golovatch et al., 2009).

Other alternative animal models for smoke exposure include lambs and primates. In young lambs morphological changes in the lung following second hand smoke exposure can appear after a short period of time (<1 month) (Stecenko et al., 1986) making it a useful model for smoke exposure studies. In addition, the lungs of lambs are still developing after birth similar to humans, and therefore the model is potentially useful for research into the effects of second hand smoke on infant lung development. Aside from the effects of cigarette smoke on the lung, lambs have also been used to study the effect of passive smoke on laryngeal chemoreflexes (St-Hilaire et al., 2010).

Non-human primate smoking models have been utilized to demonstrate the effects of environmental smoke exposure with studies focusing on infants, lung development, and pre vs. post-natal effects. This model is particularly useful for studying the effects of maternal smoke exposure during pregnancy (United States. Public Health Service. Office of the Surgeon General, 2006) as well as the carcinogenic effects of cigarette smoke (Coggins, 2001). Investigators have used a variety of smoke exposure methods with the primates including training non-human primates to puff cigarettes (Ando and Yanagita, 1981). Unfortunately the primate model is difficult to work with secondary to regularity issues and enormous costs.

The use of large animals including lambs and non-human primates offer the advantage of more closely mimicking human exposure, especially in prenatal and neonatal environments. However, the disadvantages of all of the large animal models include cost, size, and feasibility of smoke exposure studies, as well as a lack of available reagents.

## Conclusion

In conclusion, there is a substantial amount of information pertinent to second hand smoke exposure that can be gained from an understanding of animal models of disease. It is important to understand the limitations of each model of smoke exposure. Rodents, while widely used, have several limitations, including strain differences in disease susceptibility, and relatively mild forms of emphysema. However, there is significant information that can be gained from the use of mouse models, especially in an era of transgenic manipulation. The most attractive alternative smoke exposure animal models are guinea pigs and rabbits, both developing clear evidence of inflammation and emphysema. A better understanding of the benefits and drawbacks of these models will assist researchers in best choosing the appropriate species and exposure model prior to conducting smoke exposure experiments.

### Conflict of interest statement

The authors declare that the research was conducted in the absence of any commercial or financial relationships that could be construed as a potential conflict of interest.
